# Analysis of the efficacy of UBE in the treatment of tophus deposition within the lumbar spinal canal

**DOI:** 10.1097/MD.0000000000045466

**Published:** 2025-10-31

**Authors:** Yingying Qian, Jiang Liu, Mingjie Xiao, Wen Chen, Yonghui Feng, Chunliang Xie, Sineng Zhang, Wenliang Liu, Fengwei Qin

**Affiliations:** a Guangzhou Hospital of Integrated Traditional Chinese and Western Medicine Affiliated to Guangzhou University of Traditional Chinese Medicine, Guangzhou City, Guangdong Province, China; b Guangzhou Hospital of Integrated Traditional and Western Medicine, Guangzhou City, Guangdong Province, China.

**Keywords:** intraspinal tophus, UBE

## Abstract

**Rationale:**

Lumbar intraspinal gouty tophus deposition is clinically rare, with nonspecific clinical/imaging features leading to frequent misdiagnosis. Current treatments mainly rely on open surgery, and systematic reports on Unilateral Biportal Endoscopy (UBE) application are scarce, necessitating exploration of UBE’s efficacy.

**Patient concerns:**

Seven patients (October 2023–November 2024) presented with persistent low back pain, accompanied by lower limb pain and/or numbness. Conservative treatment (urate-lowering drugs, anti-inflammatory drugs, and physical therapy) for ≥3 months was ineffective, and symptoms persisted or worsened.

**Diagnoses:**

Postoperative pathological examination confirmed urate crystal deposition (gold standard for gouty tophi). Preoperative computed tomography showed spinal canal soft tissue calcification, and magnetic resonance imaging revealed lumbar facet joint amorphous substance deposition, with obvious lumbar spinal stenosis and nerve root compression.

**Interventions:**

All patients underwent UBE surgery—removing tophi, decompressing spinal/nerve root canals, placing drainage tubes, and receiving postoperative urate-lowering, anti-inflammatory, and nerve-nourishing treatments.

**Outcomes:**

All surgeries succeeded; VAS (8.00 ± 0.82→1.57 ± 0.54) and ODI (66.14 ± 3.93→18.29 ± 2.69) improved significantly (*P* < .05). No complications occurred; 3–12 months follow-up showed no tophus recurrence, and patients resumed daily activities.

**Lessons:**

UBE has good short-term efficacy for this disease, offering a minimally invasive option, but long-term efficacy requires further observation to supplement spinal gout treatment evidence.

Gout is a disease caused by purine metabolism disorders leading to elevated blood uric acid levels, which results in the deposition of urate crystals in bones, joints, tendons, ligaments, subcutaneous tissues, soft tissues, nerves, and other regions throughout the body, triggering localized sterile inflammatory reactions.^[1]^ Globally, the incidence of gout ranges from 0.1% to 0.3%,^[2]^ which is more common in adult men and postmenopausal women. The main symptoms include acute and chronic arthritis, tophi formation, joint deformity and movement disorders, etc. Tophi, a hallmark of gout, typically develop in peripheral joints.^[3]^ Lumbar intraspinal gouty tophus deposition is clinically rare, with most cases reported as isolated instances in domestic and international literature.^[4–8]^ Due to its diverse clinical presentations and nonspecific imaging features, the condition is often misdiagnosed as lumbar disc herniation, spinal tumors, or other disorders.^[9]^ Definitive diagnosis in most cases relies on intraoperative exploration combined with postoperative pathological examination, underscoring the challenges in clinical diagnosis and management. Early symptoms primarily include localized pain and nerve root irritation. As the tophus enlarges and intraspinal occupying effects worsen, secondary spinal canal stenosis may occur, leading to compression of nerve roots or the spinal cord. Severe cases may present with urinary/fecal incontinence, incomplete paraplegia, or even complete paralysis.^[10]^ Therefore, heightened clinical vigilance toward spinal gout is warranted.

In recent years, spinal endoscopic techniques have advanced rapidly, with unilateral biportal endoscopy (UBE) garnering significant attention due to its unique technical advantages. Compared to traditional single-portal endoscopy, UBE employs independent endoscopic and operative channels, allowing the use of conventional open surgical instruments while maintaining advantages such as clear visualization, flexible maneuverability, and minimally invasive efficiency. It has emerged as a critical option in minimally invasive spinal surgery.^[11]^ However, previous studies have predominantly focused on open surgeries, and there was still a lack of systematic reports on the clinical application of UBE in the treatment of a special pathological type of lumbar spinal stenosis combined with gouty tophus deposition. This study retrospectively analyzed 7 cases of spinal canal stenosis with pathologically confirmed gouty tophus deposition treated with UBE in our hospital’s Department of Spinal Surgery from October 2023 to November 2024. The aim was to evaluate the clinical value of UBE in the treatment of lumbar intraspinal gouty tophus deposition, providing clinicians with targeted surgical insights and comprehensive imaging reference guidelines. which is reported as follows:

## 1. Clinical data

### 1.1. Inclusion and exclusion criteria

Inclusion Criteria:

1. Postoperative pathological examination clearly confirmed the deposition of urate crystals (needle-shaped crystals observed under a microscope, which is the gold standard for diagnosing gouty tophus);
2. Preoperative CT and MRI examinations showed obvious spinal canal stenosis at the lumbar segment (the spinal canal area at the lesioned segment < 150 mm²) and corresponding nerve root compression signs (such as disc protrusion, ligamentum flavum thickening, or soft tissue masses compressing the nerve roots);
3. Conservative treatment (including urate-lowering drugs [such as allopurinol, febuxostat] with a dosage adjusted to maintain blood uric acid < 360 μmol/L, non-steroidal anti-inflammatory drugs [such as celecoxib] for pain relief, and physical therapy [such as lumbar traction, microwave therapy]) was ineffective for ≥ 3 months, and the clinical symptoms (low back pain, lower limb pain/numbness) persisted or aggravated;
4. The patients had complete medical records (including preoperative and postoperative imaging data, laboratory test results, and follow-up records) and could cooperate with the follow-up.

Exclusion Criteria:

1. Concurrent spinal diseases other than gouty tophus, such as spinal tumors (primary or metastatic), spinal tuberculosis, spinal infection (bacterial or fungal), or traumatic spinal injuries (such as vertebral fractures);
2. Severe systemic organic diseases that cannot tolerate surgery, such as severe heart failure (New York Heart Association [NYHA] class IV), severe liver and kidney dysfunction (liver function: alanine aminotransferase > 3 times the upper limit of normal; renal function: estimated glomerular filtration rate < 30 mL/min/1.73 m²), uncontrolled diabetes mellitus (fasting blood glucose > 13.9 mmol/L), or coagulation disorders (prothrombin time > 18 seconds, activated partial thromboplastin time > 60 seconds);
3. Spinal structural abnormalities, such as scoliosis (Cobb angle > 10°), lumbar spondylolisthesis (Meyerding grade ≥ II), or severe spinal deformities (such as kyphosis with a Cobb angle > 30°), which require fusion surgery;
4. History of previous lumbar or other spinal surgeries (such as laminectomy, discectomy, or spinal fusion), which may affect the surgical approach and efficacy evaluation;
5. Mental disorders (such as schizophrenia, severe depression) that make it impossible to accurately evaluate pain and functional status (using Visual Analogue Scale [VAS] and Oswestry Disability Index [ODI]).

### 1.2. General information

**Figure 1.**
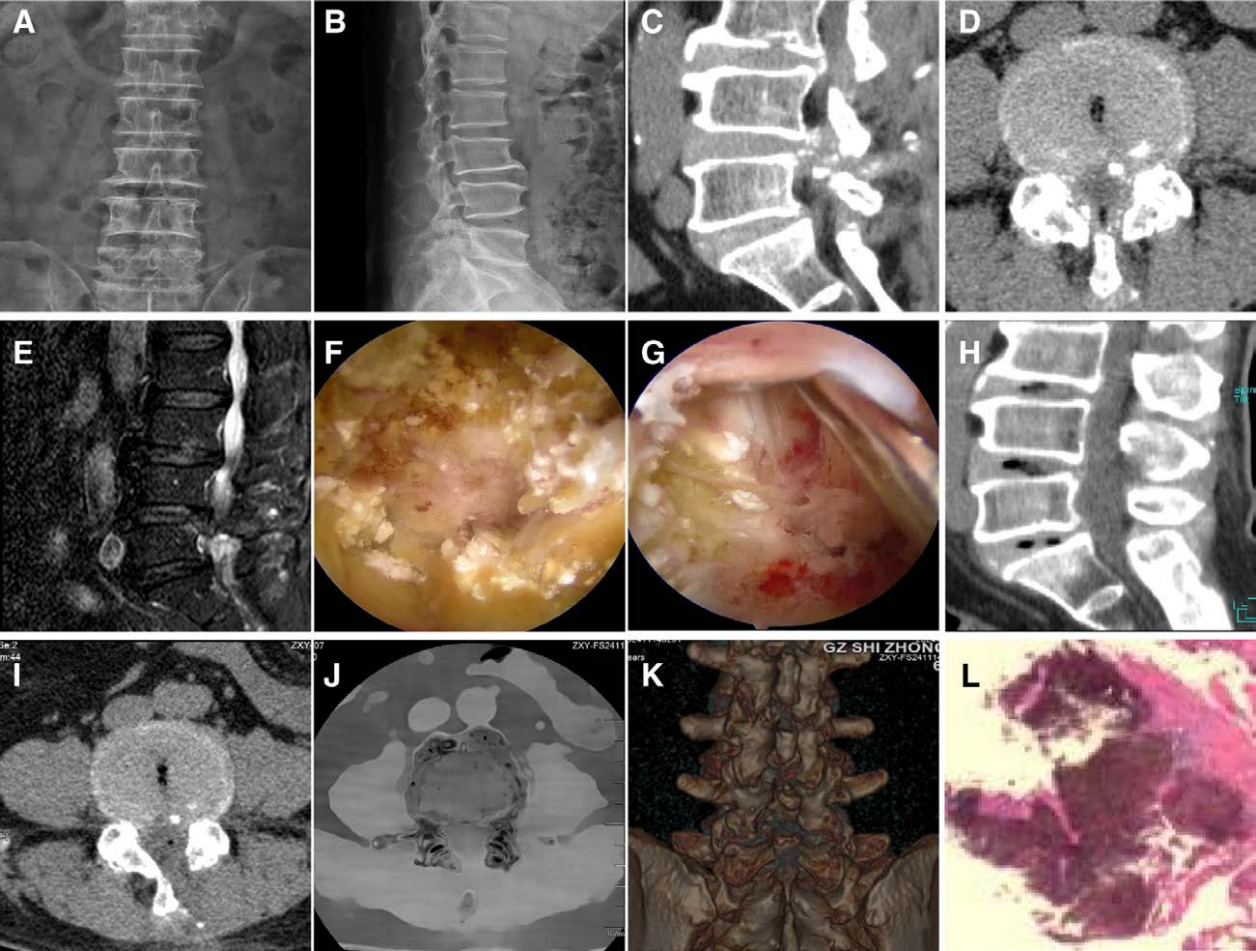
Patient, male, 68 years old, underwent endoscopic spinal surgery for L4/5 lumbar spinal stenosis with a previous history of gout, no obvious tophi and joint swelling in the limbs, and blood uric acid was 289 µmol/L.Postoperative uric acid-lowering therapy was given. (A, B) The preoperative anterior and lateral positions X-ray showed lumbar degeneration, (C, D) CT scan, L4/5 disc level ligamentum flavum hypertrophy with partial high density, (E) sagittal MRI shows L4/5 disc herniation with spinal stenosis, (F, G) intraoperative microscopy showed tophi deposited under the ligamentum flavum, around the articular eminence, in the intervertebral discs and under the posterior longitudinal ligament, (H, I) Postoperative CT scan showed that there was sufficient decompression of the spinal canal and tophi were removed, (J, K) postoperative energy spectrum analysis, the speckled green-stained areas represent gouty tophus crystal deposition. (L) Pathological examination showed that the localized area was covered with deposits of uric acid crystals, and the fibrochondral tissues showed degeneration (HE, ×200).

A total of 7 patients were included in this study, consisting of 5 males and 2 females. Their ages ranged from 62 to 76 years, with an average age of (71.00 ± 4.73) years. The clinical data of the 7 patients with tophus deposition in the lumbar spinal canal are shown in Table 1. All cases met the aforementioned criteria, with clinical manifestations including low back pain accompanied by lower limb pain and(or) numbness. The preoperative VAS for pain averaged (8.00 ± 0.82) points, and the ODI averaged (66.14 ± 3.39). CT scans revealed soft tissue calcification around the facet joints or calcification foci around the ligamentum flavum in the spinal canal (Fig. 1C,D). MRI scans could show the deposition of amorphous substances around the lumbar facet joints (Fig. 1E). Five patients had a history of gout, and 4 patients had high uric acid levels in preoperative examinations, with an average uric acid value of (458.29 ± 131.28) µmol/L, reference range is 202.00 to 416.00 µmol/L. This study was approved by the Ethics Committee of our hospital Approval No.: 20240816003. Informed consent was waived due to the retrospective nature of the analysis and anonymized data processing, in compliance with Article 21 of the Ethical Review Measures for Life Sciences and Medical Research Involving Human Subjects.

**Table 1:** Clinical data of 7 patients with tophus deposition within the spinal canal.

Patient number	Age	Sex	Main symptoms	Lesioned segments	History of gout	Uric acid level	Preoperative medication	Surgical methods
1	68	Male	Low back pain accompanied by radiating pain in the left lower limb.	L4/5	Yes	289	No	UBE
2	74	Female	Low back pain accompanied by numbness and pain in both lower limbs.	L4/5	Yes	627	No	UBE
3	62	Male	Low back pain accompanied by pain in the left lower limb.	L4/5	No	377	No	UBE
4	76	Male	Low back pain accompanied by pain in the right lower limb.	L5/S1	No	352	No	UBE
5	74	Male	Pain and numbness in the left lower limb.	L5/S1	Yes	423	No	UBE
6	72	Female	Low back pain accompanied by radiating pain in the left lower limb.	L3/4	Yes	603	Yes	UBE
7	71	Male	Low back pain accompanied by numbness and weakness in the left lower limb.	L3/4	Yes	537	No	UBE

### 1.3. Surgical methods

All surgeries were performed by the same senior spinal surgeon (attending surgeon: Associate Professor, with 10 years of surgical experience, having performed over 500 major spinal procedures to date, including an annual average of 200 surgeries over the past 2 years, of which UBE procedures comprised a significant portion). No variations in surgical outcomes attributable to technical disparities among operators were observed.

#### 1.3.1. Surgical procedure

All 7 patients underwent UBE surgery for treatment. After successful general anesthesia, the patient was placed in the prone position on a soft cushion with the waist flexed and the abdomen suspended. The affected vertebral lamina space was accurately located with the preoperative positioning guide pin, and the skin surface was marked. The responsible surgical segment was located by C-arm fluoroscopy. Then, the endoscopic system, the water injection system, and the power system were connected. Under the assistance of fluoroscopy, along the fluoroscopic marking lines, the skin and deep fascia were successively incised at the working channel (about 1.5 cm) and the viewing channel (about 1.5 cm). The 3-stage dilatation catheters were placed for soft tissue dilatation and muscle dissection, and the space was rechecked by fluoroscopy to ensure its accuracy. A 4mm UBE 30°endoscope was inserted into the viewing channel, and continuous gravity perfusion irrigation was carried out with the fluid being drained out through the working channel. The 90°radiofrequency ablation electrode was used for hemostasis and soft tissue cleaning to expose the upper and lower vertebral laminae and the ligamentum flavum of the responsible segment. The bone power system under the endoscope was used, and the vertebral lamina bone was removed by grinding and biting with the reverse vertebral rongeur. The ligamentum flavum was dissected and removed with the nerve dissector, and the herniated nucleus pulposus tissue was removed with the nucleus pulposus forceps. The areas with tophi deposition were carefully resected to completely decompress the nerve root canal. A negative pressure drainage tube was placed under the endoscope, and the incisions were sutured layer by layer and covered with sterile dressings. The tissues with tophi deposition removed from all patients were routinely sent for pathological examination after the operation. During and after the operation, the vital signs of the patients were stable, and no special discomfort was reported. The patients were safely returned to the ward.

The surgical drain was removed when the observed drainage volume was less than 20 mL/24 h after the operation. Prophylactic antibiotic treatment was administered for 24 hours. After the operation, treatments such as anti-inflammatory therapy, nerve-nourishing therapy, and drugs to promote uric acid excretion were given. The blood uric acid level was regularly rechecked, and the patients were instructed to follow a low-purine diet. The patients were allowed to get out of bed and move around with a lumbar brace 24 to 48 hours after the operation. The patients were guided to perform early functional exercises of the back and lumbar muscles. Excessive loading on the waist and vigorous exercise were to be avoided within 3 months.

### 1.4. Evaluation indicators

The content includes VAS pain score, ODI score, as well as postoperative reexamination by X-ray, CT or MRI to understand the recovery of lumbar spine function. The VAS score is a pain assessment tool. The attending doctor will explain the scoring criteria to the patient. A score of 0 indicates no pain, and a score of 10 indicates extreme pain. The patient needs to select a number within the range of 0 to 10 to represent the degree of pain according to their actual feelings.The ODI scores range from 0 to 100, with higher scores indicating more severe functional impairment.

### 1.5. Statistical methods

Data analysis was performed using SPSS 27.0 software. Measurement data were expressed as x̅±s. Paired t tests were used for comparison between the 2 groups. Chi-square test was used for count data. *P* < .05 was considered statistically significant for the difference, and calculate Cohen *d* effect size (|*d*| > 0.8 indicates a large effect).

## 2. Results

### 2.1. Clinical results

All 7 patients successfully completed the UBE surgery, with an average operation time of (85.29 ± 10.36) minutes and an average intraoperative blood loss of (35.71 ± 8.24) mL. During the operation, it was clearly observed that the herniated nucleus pulposus tissue was mixed with off-white, gritty tophi, which were mainly distributed under the ligamentum flavum, around the articular eminence, in the intervertebral disc, and under the posterior longitudinal ligament (Fig. 1G). Postoperative pathological examination showed that all specimens contained needle-shaped urate crystals (stained red by HE staining, Fig. 1L), which were consistent with the diagnosis of gouty tophus.

Compared with preoperative status, the patients’ clinical symptoms were significantly improved after operation: low back pain, lower limb radiating pain, and numbness were significantly relieved. On the 2nd day after operation, all patients could get out of bed and move around with the assistance of a lumbar brace, and the average time of getting out of bed was (36.57 ± 6.82) hours. No complications such as incision infection, cerebrospinal fluid leakage, nerve root injury, or hematoma occurred during the perioperative period.

The VAS score and ODI showed significant improvements (Table 2): the preoperative VAS score was (8.00 ± 0.82) (indicating severe pain), which decreased to (1.57 ± 0.54) (indicating mild or no pain) at the last follow-up, with a mean decrease of (6.43 ± 0.78) points; the preoperative ODI was (66.14 ± 3.93) (indicating severe functional impairment), which decreased to (18.29 ± 2.69) (indicating mild functional impairment) at the last follow-up, with a mean decrease of (47.85 ± 4.12) percentage points. The differences between preoperative and postoperative scores were statistically significant (*P* < .001), and the Cohen *d* values (5.67 for VAS and 9.36 for ODI) indicated extremely large effect sizes, confirming the significant therapeutic effect of UBE.

**Table 2:** Comparison of VAS scores and ODI before and after surgery.

Patient number	VAS score		ODI (%)	
	Preoperative	Postoperative	Preoperative	Postoperative
1	9	2	72	20
2	7	2	64	22
3	7	2	62	18
4	9	1	68	14
5	8	2	70	18
6	8	1	62	20
7	8	1	65	16
*x̅*±s	(8.00 ± 0.82)	(1.57 ± 0.54)	(66.14 ± 3.93)	(18.29 ± 2.69)
*t*-value	*t* = 15.00		*t *= 24.76	
*P*-value	*P* < .001		*P* < .001	
95% CI	(5.38–7.48)		(43.13–52.58)	
Cohen d*	5.67		9.36	

ODI = Oswestry Disability Index, VAS = Visual Analogue Scale.

* The paired *t*-test results demonstrated statistically significant improvements in both VAS and ODI scores (*P* < .001). Cohen *d* values were 5.67 (VAS) and 9.36 (ODI), both indicating extremely large effect sizes.

Postoperative follow-up lasted 3 to 12 months (average (7.57 ± 2.31) months). At the last follow-up, the patients’ low back pain and lower limb pain/numbness basically disappeared, and no recurrence of tophus, nerve function impairment, or need for reoperation was found. All patients returned to their daily activities (such as walking, housework) within 3 months after operation, but avoided heavy physical labor and strenuous exercise as recommended.

### 2.2. Imaging evaluation

Preoperative imaging showed no specific findings on X-ray (Fig. 1A, B); CT showed disc degeneration, protrusion, lateral recess stenosis at the lesioned segments, and multiple punctate high-density shadows (suggestive of tophus calcification) in the vertebral appendages and adjacent soft tissues (Fig. 1C, D); MRI showed disc protrusion, ligamentum flavum thickening, spinal canal stenosis, and low T1 signal/isosignal and high T2 signal/low signal lesions (consistent with tophus deposition) around the facet joints (Fig. 1E).

Postoperative imaging reexamination (CT and MRI) showed that the spinal canal and nerve root canal were fully decompressed: the high-density shadows of tophus on CT basically disappeared (Fig. 1H, I), and the spinal canal area at the lesioned segments increased significantly from preoperative (120.5 ± 3.3) mm² to postoperative (222.7 ± 10.2) mm², with an average increase of (84.7%±4.1%) (*P* < .001, Cohen *d* = 10.43). The tophus clearance rate was (96.43%±1.27%) (≥95%), indicating that UBE could effectively remove the lesions (Table 3). In one patient who underwent dual-source CT dual-energy spectral imaging after operation, scattered speckled green-stained areas (representing residual urate crystals) were found in the vertebral appendages and adjacent soft tissues, but the volume was small (accounting for < 5% of the original tophus volume) and did not cause clinical symptoms.

**Table 3:** Quantitative assessment of postoperative imaging.

Parameter	Preoperative	Postoperative	Percentage increase	*P*-value	95% CI	Cohen *d*
Spinal canal area (mm^2^)	120.5 ± 3.3	222.7 ± 10.2	84.7%±4.1%	<.001	(94.31–10.1)	10.43
Tophus clearance rate (%)	-	96.43%±1.27%	-	=.012	(95.25–97.61%)	1.12

## 3. Discussion

Gout is a common hereditary or acquired metabolic disorder,^[12]^ the pathogenesis of which involves reduced excretion of uric acid and impaired purine metabolism, and the attacks of the disease can involve all joints of the body, the most common first manifestation of the first metatarsophalangeal joints of the erythema, swelling and heat pain, and the incidence of spinal vertebral body involvement is extremely low, and so far, the incidence of spinal gout has been reported to be more than 130 cases at home and abroad.^[13]^ Kersley et al^[14]^ first reported a case of cervical gout in 1950, which has gradually attracted attention because of its ability to destroy spinal structures and damage nerves, with serious consequences such as claudication and paralysis. Spinal gout is essentially an inflammatory disease caused by the deposition of urate crystals (monosodium urate) in the spine.^[15]^ Epidemiologic studies have shown that the lumbar spine has the highest rate of involvement, accounting for 56% of all spinal gout cases, followed by the cervical spine, and less frequently the thoracic spine.^[16]^ The spine, as a triple-joint complex structure with a large number of ligaments and synovium, could theoretically be equally susceptible to urate deposition at these sites, which in turn may lead to a reduction in the volume of the spinal canal (neural radicular canal), causing or exacerbating spinal stenosis. It is currently believed that the pathogenic mechanism of spinal gout may be related to local blood transport disorders and local blood pH imbalance, small joint injury, and local inflammation.^[17,18]^

Spinal gout lacks specificity in clinical manifestations, imaging and laboratory tests, and is easily confused with spinal tumors, nucleus pulposus prolapse, spinal stenosis, disc calcification, etc. Thompson et al reported a case of spinal gout misdiagnosed as metastatic spinal tumor.^[19]^ Therefore, early diagnosis is important for the treatment of spinal gout. In terms of laboratory tests, most patients showed elevated blood uric acid, which is suggestive of spinal gout; however, about 20% of spinal gout patients had serum uric acid levels within the normal range.^[18]^ In this study, all of them were admitted to the hospital to receive treatment for lumbar spondylolisthesis and lumbar spinal stenosis, and 3 of them had normal preoperative uric acid and no previous history of gout, and a large number of gout stones around the ligamentum flavum were found only during the operation, and the gout stones were completely removed intraoperatively and decompression of the root canals was carried out. Imaging is a routine means to assist in the diagnosis of spinal gout,^[20,21]^ but it has limitations, with no specificity in the performance of X-ray examination; CT is superior to MRI in identifying calcified foci, which are manifested as bone destruction and erosion of the vertebral body, vertebral plate, or articular eminence, or accompanied by calcification; MRI has the advantage of soft-tissue resolution, and by combining the previous reports with the present study, spinal gout is often manifested by MRI with a low T1 signal or isosignal, and T2 is high signal or low signal. However, CT and MRI have low specificity for monosodium urate crystallization, which makes it difficult to differentiate it from other spinal space-occupying diseases. This diagnostic dilemma was particularly prominent in this study: 2 patients in this study had relatively diagnostic manifestations on preoperative CT scans, and the rest of the patients were not found to have gouty stone deposits until intraoperative lumbar root canal decompression was performed, which emphasizes the need for improved preoperative diagnostic tools and the necessity of multimodal evaluation.

With the development of imaging technology, the emergence of DECT (dual-source CT dual-energy imaging) has brought great convenience to the diagnosis of spinal gout, with high sensitivity (92%) and specificity (85%), can specifically analyze the urate component of gouty nodules, and is able to identify urate deposition thus improving the diagnostic accuracy of spinal gout.^[22,23]^ Its working principle is mainly based on the decay characteristics of atomic number, because the atomic number of calcium is higher than that of urate, resulting in the release of greater energy in the decay process, the computer will convert this difference in energy changes into different CT values, and the final output image displays urate (green) and calcium (purple) in different colors, to achieve the accurate identification and qualitative analysis of urate crystals.^[24]^ Meanwhile, it can clearly show the site, size, and shape of urate crystal deposition, which is considered a gout screening tool with unique advantages. However, at present, dual-energy CT is mainly used for the diagnosis of gout in the joints of the extremities, and relatively few studies have been conducted on the application of gout in the spine. Another study reported^[25]^ that DECT showing monosodium urate crystal signal in the spine of middle-aged and elderly men may also be seen in physiologic conditions and may not be specific for spinal gout. Although the preliminary findings suggest that DECT has great research value and application potential, its value in the diagnosis of spinal gout remains to be confirmed by further studies.

Due to the complexity and challenging nature of spinal gout disease, strict case selection and detailed preoperative evaluation is the key to ensure the efficacy of UBE surgery. The author suggests a stepwise diagnostic process: CT and MRI combined evaluation, carefully read the films preoperatively, combined with the clinical manifestations and CT, MRI, and other examinations, to determine the degree of nerve compression and details such as whether it is combined with calcified lesions, and provide individualized surgical plan; priority DECT screening for suspected cases, but be alert to false negatives at atypical sites; and intraoperative frozen pathology as the gold standard for diagnosis.

Treatment options for spinal gout vary depending on the condition. For patients with a history of gout who have no signs of nerve damage and <3 months of onset, conservative treatment is generally recommended, including following a low purine diet, pharmacologic therapy (uric acid-lowering, nonsteroidal anti-inflammatory drugs, and glucocorticoids), and lifestyle interventions. However, when patients present with significant spinal cord and nerve root compression with corresponding clinical symptoms and conservative treatment is not effective, surgical intervention should be aggressively pursued. Traditional posterior lumbar interbody fusion, as a classic procedure for lumbar degenerative diseases, effectively relieves nerve compression and rebuilds spinal stability through lesion debridement, laminectomy, and internal fixation with bone grafting. However, posterior lumbar interbody fusion is more invasive, has a longer postoperative recovery period, and may be associated with complications such as infection, hematoma, and nerve injury.^[26]^ With the development of minimally invasive endoscopic techniques in the spine and favored by clinicians and patients, endoscopic gout stone removal and decompression has become possible. Among them, UBE has made remarkable progress in clinical practice, with significant advantages such as less trauma, clear vision, more thorough decompression of the area, and faster postoperative recovery, etc. By means of minimally invasive surgery, it can minimize the damage to spinal structures, while reducing intraoperative bleeding and hospitalization time, which not only effectively relieves the symptoms of patients, but also promotes the recovery of patients’ functions.^[27]^ In this study, we chose to apply UBE for lesion debridement to achieve the goal of removing the gout stone and adequately decompressing and enlarging the spinal canal, while preserving the integrity of the posterior spinal structures to a certain extent, with less impact on the stability of the spine. It has been reported in the literature^[28]^ that the UBE technique has successfully achieved extensive removal of gout stone lesions, with significant relief of postoperative lumbar pain and radiating pain in the lower extremities of the patients, as well as effective improvement of myasthenia gravis with neurological deficits, and significant reduction of intraoperative blood loss and shortening of postoperative hospitalization, which has further verified the universality of this procedure in the treatment of spinal gout.

UBE and percutaneous transforaminal endoscopic discectomy (PTED), as minimally invasive surgical options for the treatment of lumbar spinal stenosis, can both achieve significant clinical efficacy in lumbar spinal decompression. Current research has shown that UBE surgery has the following core advantages: through percutaneous minimally invasive incision, the use of multifidus interspace blunt separation to establish a working channel, effectively reducing muscle tissue damage; compatible with traditional instruments and endoscopic instruments, endoscopic magnification of the field of view to achieve a wide and clear surgical field and flexible operating space, effectively improve decompression and shorten the operating time; dual-channel independent mode of operation to break through the limitations of single-channel field of view, endoscopic, and endoscopic operation to reduce the time of operation. The dual-channel independent operation mode breaks through the limitation of single-channel field of vision, and the endoscope and instruments can be adjusted in multiple axial directions to realize the all-round decompression of the spinal canal; and the water-mediated environment can effectively control the blood seepage in the operation field, and significantly improve the efficiency of hemostasis. In contrast, PTED is limited by the inherent shortcomings of single-channel endoscopic technology, including a relatively narrow field of view, high requirements for operating techniques, special instruments, and difficulties in contralateral decompression, which reduce the efficiency of the operation to a certain extent. Nevertheless, PTED has unique advantages: it can be operated under local anesthesia, whereas UBE mostly requires general anesthesia or epidural anesthesia^[29]^; it is less locally invasive, interferes less with the intravertebral structures, and does not require indwelling drainage tubes; and it is relatively cheaper in hospitalization costs.^[30]^ Both procedures are considered to have a steep learning curve, and beginners need to practice with a large number of cases to gradually increase their proficiency and achieve the desired outcome. This suggests that when choosing a surgical procedure, clinicians should make a comprehensive analysis based on their own technical proficiency and the characteristics of the patient’s condition, so as to develop an individualized optimal treatment plan.

In summary, spinal gout faces greater challenges in preoperative confirmation of diagnosis due to the lack of typical clinical manifestations and specific imaging features. At present, pathological examination is still the gold standard for diagnosis of spinal gout stone, but the following diagnostic clues should be emphasized in clinical practice: for the complaint of low back pain, regardless of the presence of neurological impairment, if there is a previous history of gout or hyperuricemia, we should be alert to the possibility of spinal gout. In terms of imaging evaluation, DECT can be an important complementary diagnostic and therapeutic tool, but it has yet to be further promoted and applied in the clinic. The results of this study showed that UBE has diagnostic and therapeutic advantages, and is one of the therapeutic means as a treatment for gout stone deposition in the lumbar spinal canal, with the advantages of less trauma, clear field of view, faster recovery, and better short-term prognosis. For postoperative management, standardized uric acid-lowering medication combined with low purine dietary control is a key measure to prevent recurrence. Based on the systematic summary of this study, we suggest that clinicians should: improve the ability to recognize the clinical manifestations of spinal gout and establish a standardized diagnostic pathway of “history inquiry-imaging screening-pathology confirmation”; incorporate DECT into the routine preoperative examination sequence to improve the early diagnosis rate; give priority to minimally invasive procedures, such as UBE, for those who are eligible for the indications; and establish a standardized postoperative management model to ensure long-term control of uric acid levels. Only through the combination of early diagnosis, precise treatment and systematic management can the long-term prognosis of spinal gout patients be effectively improved.

This study has several limitations that should be considered when interpreting the results. First, it is a single-center retrospective study with a small sample size (only 7 cases). Lumbar intraspinal gouty tophus deposition itself is a rare disease, which leads to difficulty in expanding the sample size in a short time, and the small sample may reduce the statistical power and limit the generalization of the results. Second, the follow-up period is relatively short (3–12 months), so we can only evaluate the short-term efficacy of UBE, and cannot fully confirm its long - term effects, such as the long-term stability of the lumbar spine, the risk of tophus recurrence, and the long-term improvement of nerve function. Third, due to the retrospective design, there may be selection bias. For example, the inclusion of patients depends on the completeness of medical records, which may exclude some eligible cases with incomplete data. Fourth, this study did not set up a control group (such as patients treated with open surgery or PTED), so it is impossible to compare the advantages and disadvantages of UBE with other surgical methods in terms of long-term efficacy, complication rates, and recovery time. Fifth, the study did not conduct a comprehensive analysis of the factors affecting the efficacy, such as the relationship between the size and location of tophus and the surgical effect, and the impact of postoperative uric acid control level on the recurrence rate. In future research, we will carry out multi-center, prospective cohort studies with larger sample sizes, set up appropriate control groups, extend the follow-up time, and explore the influencing factors of efficacy, so as to provide more reliable evidence for the clinical application of UBE in the treatment of lumbar intraspinal gouty tophus deposition.

## Author contributions

**Data curation:** Jiang Liu, Mingjie Xiao, Sineng Zhang, Wenliang Liu, Fengwei Qin.

**Conceptualization:** Wen Chen, Yonghui Feng, Fengwei Qin.

**Funding acquisition:** Wen Chen, Fengwei Qin.

**Investigation:** Yingying Qian, Jiang Liu, Chunliang Xie, Fengwei Qin.

**Methodology:** Yonghui Feng, Chunliang Xie, Sineng Zhang, Fengwei Qin.

**Project administration:** Fengwei Qin.

**Resources:** Chunliang Xie, Fengwei Qin.

**Software:** Yingying Qian, Jiang Liu, Wenliang Liu.

**Supervision:** Sineng Zhang, Fengwei Qin.

**Visualization:** Sineng Zhang.

**Writing – original draft:** Yingying Qian, Fengwei Qin.

**Writing – review & editing:** Fengwei Qin.
